# A sequential allocation study to determine the ED_50_ of Dexmedetomidine as an adjuvant to lidocaine intravenous regional anesthesia

**DOI:** 10.1186/s12871-022-01702-9

**Published:** 2022-05-27

**Authors:** Cynthia Karam, Sadek Al Assadi, Ghassan Kanazi, Carine Zeeni

**Affiliations:** Department of Anesthesiology and Pain Management, American University of Beirut Medical Center, PO-Box: 11-0236, Beirut, 1107 2020 Lebanon

**Keywords:** Bier block, Dexmedetomidine, Tourniquet pain, Dixon up-and-down method

## Abstract

**Background:**

Intravenous regional anesthesia is an easy and reliable anesthetic technique, but its use is limited by tourniquet pain. Clonidine is effective in overcoming this shortcoming when used with intravenous regional anesthesia at a dose of 1 μg/kg. Dexmedetomidine has also been used successfully at a dose of 0.5 μg/kg.

**Objective:**

Based on the potency ratios of clonidine and dexmedetomidine (8 to 1) we hypothesize that a lower dexmedetomidine dose would provide patients with 50 min of pain free tourniquet time.

**Methods:**

After informed consent, patients received intravenous regional anesthesia with lidocaine and dexmedetomidine following a sequential allocation scheme. The first patient received a dose of 0.5 μg/kg of dexmedetomidine. The dose was then adjusted in 0.1 μg/kg gradients for the following patients depending on the success of the previous block. If a patient experienced tourniquet pain prior to 50 min, the next patient received a higher dose. If not, the dose was decreased. Recruitment continued until 6 independent crossovers were observed with a minimum of 20 patients. The median effective dose ED_50_ of dexmedetomidine was calculated using the modified up-and-down method.

**Main outcome measures:**

The median effective dose of dexmedetomidine (ED_50_) that provides 50 min of tolerance to the tourniquet during a lidocaine intravenous regional anesthesia by a sequential Dixon up-and-down allocation study.

**Results:**

The ED_50_ of dexmedetomidine that provided 50 min of tolerance to the tourniquet was 0.30 ± 0.06 μg/kg.

**Conclusion:**

We determined that the dexmedetomidine dose necessary to provide 50 min of pain free tourniquet time during intravenous regional anesthesia was higher than expected based on the relative alpha-2 adrenergic receptor selectivity of dexmedetomidine compared to clonidine.

**Trial registration:**

Clinicaltrials.gov: Retrospectively registered (NCT05342870; registration date: 25/04/2022).

## Introduction

Intravenous regional anesthesia or Bier block was first introduced more than a hundred years ago by August Bier [1]. Since then this anesthetic technique has gained popularity particularly because of its ease of administration and reliability in the outpatient setting [2]. Its use however is limited by the presence of tourniquet pain before the end of the procedure, the risk of local anesthesia toxicity, and the inability to provide postoperative analgesia after tourniquet release.

Many pharmacologic agents have been added to the local anesthetics of the Bier block to decrease tourniquet pain and improve postoperative analgesia with varying degrees of success. These adjuncts include but are not limited to opioids [3, 4], non-steroidal anti-inflammatory agents [5], hypnotics [6] and alpha-2 adrenergic receptor agonists [7–14]. Αlpha-2 adrenergic receptor agonists have been the focus of interest in intravenous regional anesthesia for their sedative, analgesic, and perioperative sympatholytic properties as well as their ability to prolong analgesia time when used during regional nerve blocks. Studies conducted with clonidine as an adjunct to intravenous regional anesthesia have proven its effectiveness in delaying the onset of tourniquet pain, reducing its intensity, and improving postoperative analgesia [6–8, 12]. Dexmedetomidine is a more recent alpha-2 adrenergic receptor agonist that is approximately 8 times more selective peripherally towards the alpha-2 adrenergic receptors than clonidine [14, 15]. Based on Memis’ data, tourniquet pain occurs 32 ± 10 min (95% CI 12.4 to 51.6 min) after inflation of the distal tourniquet in a lidocaine Bier block without adjuvants [8]. Furthermore, in his study when he added dexmedetomidine 0.5 μg/kg to the lidocaine tourniquet pain was delayed till 53 ± 10 min. Since the potency ratio of alpha-2 adrenergic receptor selectivity between clonidine and dexmedetomidine is 8 to 1, this would suggest that if 1 μg/kg of clonidine is effective [6, 7], then 0.125 μg/kg of dexmedetomidine would be effective, a 75% reduction in the required dose.

The aim of this double blind non-randomized Dixon up-and-down sequential allocation design study was to determine the median effective dose of dexmedetomidine (ED_50_) that provides 50 min of tolerance to the tourniquet during a lidocaine Bier block with dexmedetomidine as an adjuvant.

## Materials and methods

Ethics approval for this study (ANES.CZ.01) was provided by the American University of Beirut Institutional Review Board, Beirut, Lebanon (Chairperson Dr. Ibrahim Salti) on August 13, 2012. After securing approval, American Society of Anesthesiologists physical status (ASA) I or II patients aged 18 to 70 years old, scheduled electively to undergo unilateral minor forearm and hand surgery (carpal tunnel or tendon release/transfer) at the American University of Beirut Medical Center (AUBMC) were approached to participate in this study. Patients with Raynaud’s disease, sickle cell anemia, heart block, allergy to any of the anesthetic drugs used, and weight greater than 100 kg were excluded as were patients on alpha adrenergic agonists, with ASA physical status III or IV, or undergoing emergency surgery or pregnant patients. This study was not registered prospectively on clinical trials.gov as data collection took place from 2012 to 2013 at a time where trial registration was not mandatory. The study was retrospectively registered with clinicaltrias.gov (NCT05342870) Once written informed consent was obtained 20 patients were recruited to participate in the study and trained to use the numeric rating scale (NRS) for reporting pain intensity from 0, indicating no pain at all, to 10, indicating the worst pain imaginable.

Prior to establishing the block patients were taken to the induction room where two venous cannulas were placed: one 22-gauge cannula was placed in a vein in the dorsum of the operative hand for the block and the other one was placed in the opposite hand for crystalloid infusion and systemic intravenous (IV) drug administration (the size of the catheter was left to the discretion of the anesthesiologist). Two mg of midazolam were administered at this time. Once in the operating theatre, standard ASA monitors were placed on the patients and all of them received 3 L/min of supplemental oxygen via nasal cannula or face mask.

The operative arm was elevated for 3 min and then exsanguinated using a 10 cm Esmarch bandage and a double pneumatic tourniquet was placed around the upper arm. The proximal cuff was inflated to 250 mmHg. Circulatory isolation of the arm was verified by the absence of a pulse. Intravenous regional anesthesia was achieved using 3 mg/kg of actual body weight of lidocaine diluted with saline to a total volume of 40 ml together with the study drug. The solution was injected over 90 seconds by a blinded anesthesiologist with constant monitoring for signs and symptoms of toxicity. Once the injection was completed the 22-gauge cannula was removed and a small dressing applied. After sensory and motor block were achieved (verified 5 min after the injection) the distal tourniquet cuff was inflated at 250 mmHg followed by the release of the proximal tourniquet cuff. The patient’s arm was then surrendered to the surgical team. Time 0 (T_0_) was defined by the inflation of the distal tourniquet cuff. Time 1 (T_1_) was defined by the deflation of the tourniquet at the end of the procedure and Time 2 (T_2_) as time of arrival at the post-operative care unit (PACU).

Once the surgical procedure was completed the tourniquet was deflated cyclically (deflation for a few seconds then reinflation to minimize local anesthesia toxicity) and the patient was transferred to the PACU for monitoring. Patients were excluded if tourniquet times were shorter than 50 min.

Tourniquet pain scores were assessed and recorded every 15 min from T_0_ until T_1_ by the blinded anesthesiologist using the NRS. Boluses of fentanyl 50 μg were administered as rescue analgesics when NRS score was more than 3. The time to the first fentanyl dose was recorded as were the NRS score at the first fentanyl dose and the total amount of fentanyl used throughout the procedure. All measurements were recorded by a blinded anesthesiologist unaware of the drug allocation scheme.

Following a sequential allocation scheme, the dexmedetomidine dose for each patient was determined by the Dixon up-and-down method. The dexmedetomidine dose for the first patient was 0.5 μg/kg, which according to Memis et al. is sufficient to provide a mean of 53 min of analgesia [8] so the minimum tourniquet time chosen for our study was 50 min. The dose was then adjusted in 0.1 μg/kg gradients for the following patients depending on the success of the previous patient’s block. If a patient experienced tourniquet pain prior to T_0_ + 50 min the next patient received a higher dose. If a patient did not experience pain at T_0_ + 50 min of tourniquet time the dose for the following patient was decreased. Identical syringes containing the solution mix were prepared by the principal investigator and handed to the blinded anesthesiologist in the room. Stopping rules were set at 6 independent crossovers in the same direction (no pain to pain or pain to no pain), with a minimum number of 20 patients. The mean and standard deviation of the ED_50_ of dexmedetomidine were calculated using the modified up-and-down method, which uses the average dexmedetomidine dose of all independent pairs of patients involved a crossover to calculate the mean and the standard deviation of the ED_50_ (main outcome). Basic demographic data recorded included sex, age, height, weight, ASA physical status and type of surgery. Data recorded in the PACU included degree of sedation measured by the modified Observer’s Assessment of Alertness/Sedation score (OAA/S) score, NRS pain scores, blood pressure and heart rate measurements recorded every 15 min for the first hour then every 30 min until discharge in addition to time to first analgesic requirement in PACU, NRS at that time, and any local or systemic complication arising throughout the study period. In addition, demographic and clinical data were analyzed comparing patients who experienced tourniquet pain versus those who did not with non-parametric tests, namely Mann Whitney U test for continuous data and chi-square test for categorical data. Statistical significance was set at *P* values less than 0.05 and results were analyzed using non-parametric tests with IBM SPSS Statistics for Windows, version 27 (IBM Corp., Armonk, N.Y., USA).

## Results

**Fig. 1:**
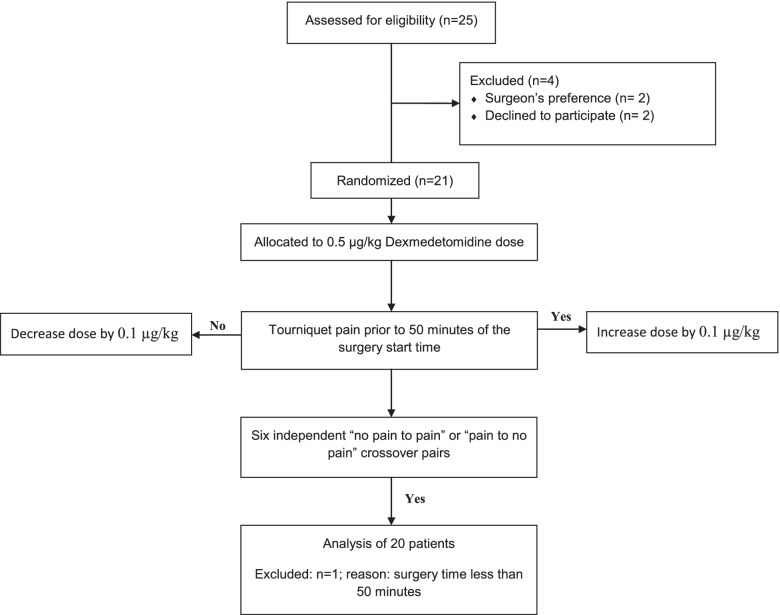
Flow diagram for the Dixon’s up-and-down method

**Fig. 2:**
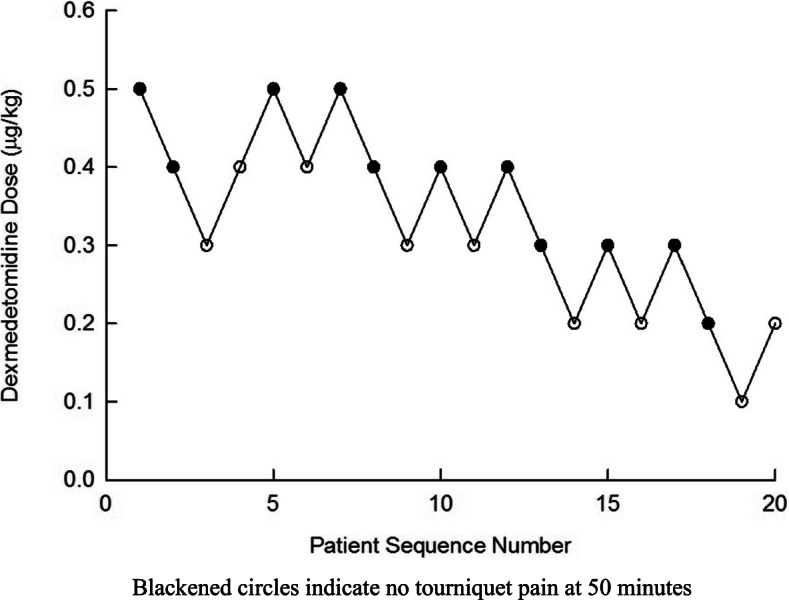
Up-and-down oscillation curve of dexmedetomidine dose added to lidocaine intravenous regional anesthesia

Twenty-five patients were assessed for study eligibility and 23 were approached. Recruitment took place over a one-year period during the year 2012–2013. Twenty-one patients provided informed consent; one patient was excluded when the surgery was completed in less than 50 min of tourniquet time. A flow diagram for the Dixon’s up-and-down method is presented in Fig. 1. Demographic and clinical results are displayed in Table 1. The up-and-down oscillation curves are illustrated in Fig. 2. The ED_50_ of dexmedetomidine was 0.30 ± 0.06 μg/kg according to the up-and-down method. None of the patients experienced hypoxemia (O_2_ saturation less than 93%), hypotension (mean arterial pressure less than 80% of baseline), bradycardia (heart rate less than 55 bpm) or other signs of local anesthesia toxicity. During their stay in the PACU none of the patients experienced marked sedation. Comparative analysis between groups revealed that patients having experienced tourniquet pain had higher systolic and diastolic blood pressures at all times and had higher NRS pain scores at T_0_ + 30 and T_0_ + 45 min. There were no differences between groups for the other variables. The dose used by Memis et al [8] corresponds to approximately ED_83_ in our data.

**Table 1:** Demographic and clinical data

	All patients	Pain	No Pain	*P* value
Sex (females: males)	16 (80%)/ 4 (20%)	6 (67%)/ 3 (33%)	10 (91%)/ 1 (9%)	0.3
Age (years)	49 ± 20	56 (42–69)	35 (24–67)	0.2
Height (cm)	164 ± 11	160 (153–172)	163 (157–173)	0.8
Weight (kg)	72 ± 14	65 (62–89)	64 (62–73)	0.8
American Society of Anesthesiologists Physical Status I/II	10 (50%)/ 10 (50%)	4 (44%)/ 5 (56%)	6 (55%)/ 5 (45%)	1.0
Type of Surgery				0.7
Ganglion surgery	6 (30%)	3 (33%)	3 (27%)	
Carpal tunnel release	8 (40%)	3 (33%)	5 (46%)	
Tendon release/transfer	5 (25%)	2 (22%)	3 (27%)	
Combination tendon release/ganglion surgery	1 (5%)	1 (11%)	0 (0%)	
Time t_0_ to t_1_ (minutes)	52 ± 4	50 (50–55)	50 (50–50)	0.8
First Fentanyl Dose (μg)^a^				
Time to first fentanyl dose (minutes)	29 (5 to 45)	–	–	–
Numerical Rating Scale (0 to 10) Score at first fentanyl dose	5.5 (3 to 9)	–	–	–
Total fentanyl dose (μg)	50 (25 to 200)	–	–	–
Numerical Rating Scale (0 to 10) Score				
T_0_	0 (0 to 2)	0 (0–1)	0 (0–0)	0.6
T_0_ + 15 min	0 (0 to 4)	2 (0,3)	0 (0–1)	0.1
T_0_ + 30 min	1 (0 to 8)	3 (2,5)	0 (0–0)	0.004
T_0_ + 45 min	1.5 (0 to 9)	5 (3,7)	0 (0–1)	0.001
Systolic Blood Pressure (mm Hg)				
T_0_	136 ± 21	145 (139–160)	118 (113–131)	0.003
T_0_ + 15 min	135 ± 21	145 (134–160)	115 (110–132)	0.01
T_0_ + 30 min	136 ± 23	147 (130–166)	117 (110–137)	0.02
T_0_ + 45 min	135 ± 22	145 (126–158)	124 (113–130)	0.02
Diastolic Blood Pressure (mm Hg)				
T_0_	77 ± 14	85 (78–94)	73 (64–77)	0.01
T_0_ + 15 min	75 ± 13	88 (77–91)	68 (62–74)	0.01
T_0_ + 30 min	74 ± 14	82 (77–91)	72 (61–75)	0.05
T_0_ + 45 min	76 ± 13	84 (73–96)	73 (62–80)	0.05
Heart Rate (beats per minute)				
T_0_	75 ± 13	68 (65–79)	77 (70–85)	0.3
T_0_ + 15 min	71 ± 14	62 (58–68)	75 (67–79)	0.1
T_0_ + 30 min	73 ± 12	67 (62–73)	78 (66–82)	0.2
T_0_ + 45 min	69 ± 12	64 (59–68)	71 (62–81)	0.1

Data are N (%), median (range) or mean ± S.D

^a^A total of 10 fentanyl doses was given to patients who experienced tourniquet pain

## Discussion

Tourniquet pain is described as a dull achy pain that causes increasing levels of discomfort over time at the level of the tourniquet despite adequate intravenous regional anesthesia. This pain is relieved immediately upon deflation of the tourniquet but is replaced with reperfusion and surgical site pain if more long-term analgesia has not been provided prior to deflation [7, 16]. The exact etiology of tourniquet pain is unclear, but multiple mechanisms have been proposed including nerve ischemia and compression [17, 18]. These noxious stimuli activate unmyelinated C fibers responsible for dull achy pain as well as smaller myelinated a-delta fibers directly under or close to the edge of the tourniquet [16, 18]. These findings shed light on the relative failure of opioids as adjuncts in intravenous regional anesthesia as demonstrated by the meta-analysis conducted by Picard [19].

Alpha-2 adrenergic agonists are widely used in anesthesia for their sedative, analgesic, anti-emetic, and anesthesia sparing properties. They have been utilized in particular as adjuncts in regional anesthesia such as intrathecal, caudal and peripheral nerve blocks to prolong anesthesia duration and improve the quality of the blocks [20–23]. Lurie et al demonstrated that adding 1 μg/kg of clonidine to lidocaine intravenous regional anesthesia in healthy volunteers delayed the onset of severe tourniquet pain [7]. These results echo Gorgia’s study [6] that used the same clonidine dose of 1 μg/kg and Gentili’s work [10] who showed that 150 μg of clonidine added to lidocaine intravenous regional anesthesia dramatically improved tourniquet tolerance and recommended this combination whenever long tourniquet times were expected. Reuben and colleagues demonstrated that adding 1 μg/kg of clonidine to the lidocaine intravenous regional solution improved postoperative analgesia as evidenced by lower pain scores and lower analgesic consumption [12]. This clonidine-induced prolongation of postoperative analgesia was not reproduced in either Gentili’s study [10] or Kleinschmidt’s work that added clonidine to prilocaine intravenous regional anesthesia [11]. Side effects after cuff deflation when using clonidine as an adjunct included hypotension and sedation in some studies [10, 11] but was better tolerated in others [6, 12].

Three studies used dexmedetomidine as an adjuvant to lidocaine in intravenous regional anesthesia. Esmaoglu used 1 μg/kg of dexmedetomidine added to lidocaine in his prospective randomized trial and found that the combination improved the quality of the anesthetic and decreased analgesic requirements intraoperatively and postoperatively without tackling the issue of tourniquet pain explicitly [9]. Memis used half the dose of dexmedetomidine and reported improved quality of anesthesia, perioperative analgesia, and prolonged tolerance to the tourniquet without any side effects [8]. Lastly Abosedira compared 1 μg/kg of clonidine and 1 μg/kg of dexmedetomidine head-to-head as lidocaine intravenous regional anesthesia adjuvants in his prospective randomized trial and concluded that dexmedetomidine provided significantly better quality of anesthesia and perioperative analgesia compared with clonidine but with slightly more postoperative sedation [13].

Our study found that the dose chosen by Memis corresponds to approximately ED_83_ in our data as the dose necessary to permit tolerance to a tourniquet time of 50 min. This dose is higher than what we expected based on the relative alpha-2 adrenergic receptor selectivity of dexmedetomidine compared to clonidine. Alpha-2 adrenergic receptors are ubiquitous in the body and are present in the central nervous system, sympathetic peripheral nerves, and vascular smooth muscles among other places. Although alpha-2 adrenergic receptors located at nerve endings may have a role in the analgesic effect of clonidine through a decrease in peripheral perineural inflammation, this mechanism of action seems less likely in our patient population given the relatively long time necessary to produce this effect. Clonidine has been found to prolong the action of local anesthetics through alpha-2 adrenergic receptor mediated vasoconstriction and subsequent decreased local anesthetic systemic absorption [24]. This vasoconstriction seems to be more pronounced with dexmedetomidine [25], and while valid for perineural administration in the case of intravenous regional anesthesia this mechanism of action seems implausible. Direct enhancement of peripheral lidocaine analgesic action by dexmedetomidine through alpha-2 adrenergic receptor action inhibiting norepinephrine release has been shown by Yoshitomi [26]; this mechanism however does not seem to be the primary purveyor of analgesia when using alpha-2 adrenergic agonists in intravenous regional anesthesia since in our study it did not translate to a reduction of the dose of dexmedetomidine according to the comparative alpha-2 adrenergic selectivity. Previous research has demonstrated that peripheral perineural clonidine enhances post-operative analgesia through inhibition of afferent impulse conduction, especially in C fibers, by a mechanism independent of the simulation of alpha-2 adrenergic receptors [27]. This alpha-2 adrenergic independent mechanism could explain the results of our study.

## Conclusions

The ED_50_ of dexmedetomidine that provides 50 min of tolerance to the tourniquet during lidocaine intravenous regional anesthesia was 0.3 μg/kg which is higher than we expected based on the relative alpha2-adrenergic receptor selectivity of dexmedetomidine compared to clonidine. Precise determination of the clinically relevant ED_95_ of dexmedetomidine as an adjuvant to intravenous regional anesthesia to increase tourniquet tolerance time to 50 min should be undertaken using the Dixon up-and-down method with a biased coin design.

## Data Availability

The datasets used and/or analyzed during the current study are not publicly available because the publicity policy is not yet generated by our institutional review board but are available from the corresponding author on reasonable request.
